# Clinical effectiveness of Invisalign® orthodontic treatment: a systematic review

**DOI:** 10.1186/s40510-018-0235-z

**Published:** 2018-09-28

**Authors:** Aikaterini Papadimitriou, Sophia Mousoulea, Nikolaos Gkantidis, Dimitrios Kloukos

**Affiliations:** 1 Department of Orthodontics and Dentofacial Orthopedics, 251 Hellenic Air Force General Hospital, P. Kanellopoulou 3, 11525 Athens, Greece; 2 Department of Orthodontics, University Hospital Ghent P8, University of Ghent, C. Heymanslaan 10, B-9000 Ghent, Belgium; 3 Department of Orthodontics and Dentofacial Orthopedics, University of Bern, Freiburgstrasse 7, CH-3010 Bern, Switzerland

**Keywords:** Orthodontics, Invisalign, Aligner, Clinical efficiency

## Abstract

**Background:**

Aim was to systematically search the literature and assess the available evidence regarding the clinical effectiveness of the Invisalign® system.

**Methods:**

Electronic database searches of published and unpublished literature were performed. The reference lists of all eligible articles were examined for additional studies. Reporting of this review was based on the Preferred Reporting Items for Systematic Reviews and Meta-Analyses (PRISMA) guidelines.

**Results:**

Three RCTs, 8 prospective, and 11 retrospective studies were included. In general, the level of evidence was moderate and the risk of bias ranged from low to high, given the low risk of bias in included RCTs and the moderate (*n* = 13) or high (*n* = 6) risk of the other studies. The lack of standardized protocols and the high amount of clinical and methodological heterogeneity across the studies precluded a valid interpretation of the actual results through pooled estimates. However, there was substantial consistency among studies that the Invisalign® system is a viable alternative to conventional orthodontic therapy in the correction of mild to moderate malocclusions in non-growing patients that do not require extraction. Moreover, Invisalign® aligners can predictably level, tip, and derotate teeth (except for cuspids and premolars). On the other hand, limited efficacy was identified in arch expansion through bodily tooth movement, extraction space closure, corrections of occlusal contacts, and larger antero-posterior and vertical discrepancies.

**Conclusions:**

Although this review included a considerable number of studies, no clear clinical recommendations can be made, based on solid scientific evidence, apart from non-extraction treatment of mild to moderate malocclusions in non-growing patients. Results should be interpreted with caution due to the high heterogeneity.

**Electronic supplementary material:**

The online version of this article (10.1186/s40510-018-0235-z) contains supplementary material, which is available to authorized users.

## Background

Orthodontic developments, especially during the last years, have been accompanied by a significant increase in the esthetic demands of the patients. Patients often express the need to influence, or even determine, treatment aspects or objectives, along with the orthodontist, driven by the effects that orthodontic appliances have in their appearance. Conventional orthodontic methods have been associated with a general compromise in facial appearance [1] raising a major concern among patients seeking orthodontic treatment [2]. Thus, esthetic materials and techniques have been introduced in clinical practice aiming to overcome these limitations [3].

Since its development in 1997, Invisalign® technology has been established worldwide as an esthetic alternative to labial fixed appliances [4–7]. CAD/CAM stereolithographic technology has been used to forecast treatment outcomes and fabricate a series of custom-made aligners using a single silicone or digital impression [6]. After its introduction, the system has been drastically developed and continually improved in many aspects; different attachment designs, new materials, and new auxiliaries, such as “Precision Cuts” and “Power Ridges” were designed to enable additional treatment biomechanics. According to the manufacturer, Invisalign® can effectively perform major tooth movements, such as bicuspid derotation up to 50° and root movements of upper central incisors up to 4 mm [8]. Despite the advocated efficiency of the treatment, its clinical potency still remains controversial among professionals, with advocates being convinced by the successfully demonstrated treated cases, as indicated by clinical evidence, in contrast to opponents who argue about significant limitations, especially in the treatment of complex malocclusions [5, 9–11].

Despite the available body of literature pertaining to Invisalign® technology, its clinical performance has been analyzed less thoroughly and a synthesis of the results still remains vague. Four systematic reviews about clear aligners exist in the literature: the first one was published back in 2005 and assessed the treatment effects of Invisalign; it included, nevertheless, only two studies [12]. More recently, another three reviews have been published. The first one was last updated in June 2014; it included 11 studies and evaluated the control of the clear aligners on orthodontic tooth movement [13]. The second one evaluated the periodontal health during clear aligner therapy and was published in the same year [14], and the most recent one was undertaken in October 2014 and included four studies, since it focused on the comparison between clear aligners and conventional braces [15].

Therefore, the purpose of the present review was to systematically search the literature and summarize the current available scientific evidence regarding the clinical effectiveness of the Invisalign® system as principal orthodontic therapy to orthodontic patients of any age treated with this method comparing either among them or those with conventional braces and evaluating the level of efficacy in various malocclusions.

## Materials and methods

### Types of studies

Randomized clinical trials (RCTs), controlled clinical trials (CCTs), and prospective and retrospective studies were considered eligible for inclusion in this review. These studies concerned to the clinical part of treatment with Invisalign, with no restrictions in language, age, status of publication, and cases with teeth extractions.

### Types of participants

Orthodontic patients of any age who were treated with Invisalign® either as the intervention or as the control group.

### Types of interventions

Invisalign® therapy. All other aligner systems have been excluded.

### Outcome

Any effect on clinical efficiency, effectiveness, treatment outcomes, movement accuracy, or predicted tooth movement in ClinCheck® of Invisalign® treatment, including changes in alignment or occlusion, treatment duration, and completion rate, as primary outcomes. Adverse events/unwanted effects have also been recorded.

### Search methods for identification of studies

Detailed search strategies were developed and appropriately revised for each database, considering the differences in controlled vocabulary and syntax rules. The following electronic databases were searched: MEDLINE (via Ovid and PubMed, Appendix, from 1946 to August 28, 2017), Embase (via Ovid), the Cochrane Oral Health Group’s Trials Register, and CENTRAL.

Unpublished literature was searched on ClinicalTrials.gov, the National Research Register, and Pro-Quest Dissertation Abstracts and Thesis database.

The search attempted to identify all relevant studies irrespective of language. The reference lists of all eligible studies were examined for additional studies.

### Selection of studies

Study selection was performed independently and in duplicate by the first two authors of the review, who were not blinded to the identity of the authors of the studies, their institutions, or the results of their research. Study selection procedure was comprised of title-reading, abstract-reading, and full-text-reading stages. After exclusion of not eligible studies, the full report of publications considered eligible for inclusion by either author was obtained and assessed independently. Disagreements were resolved by discussion and consultation with the third and the last author. A record of all decisions on study identification was kept.

### Data extraction and management

The first two authors performed data extraction independently and in duplicate. Disagreements were resolved by discussion or the involvement of two collaborators (third author and last author). Data collection forms were used to record the desired information. The following data were collected on a customized data collection form:

- Author/title/year of study
- Design/setting of the study
- Number/age/gender of participants
- Intervention and comparator/treatment duration
- Type of clinical outcome
- Method of outcome assessment

### Measures of treatment effect

For continuous outcomes, descriptive measures, such as mean differences and standard deviations, were used to summarize the data from each study. For dichotomous data, number of participants with events and total number of participants in experimental and control groups were analyzed.

### Unit of analysis issues

In all cases, the unit of analysis was the patient.

### Dealing with missing data

We contacted study authors per e-mail to request missing data where necessary. In case of no response or no provision of the missing data, only the available reported data were analyzed.

### Data synthesis

A meta-analysis was planned only if there were at least two studies of low or unclear risk of bias, reporting similar comparisons, and similar outcomes at similar time points. Otherwise, qualitative synthesis of the included studies would be performed.

### Quality assessment of included studies

The risk of bias for RCT studies was assessed by two review authors, independently and in duplicate, using the Cochrane risk of bias tool [16].

Risk of bias was assessed and judged for seven separate domains.

1. Sequence generation: was the allocation sequence adequately generated?
2. Allocation concealment: was allocation adequately concealed?
3. Blinding of participants and investigators: was knowledge of the allocated intervention adequately prevented during the study?
4. Blinding of outcome assessors: was knowledge of the allocated intervention adequately prevented before assessing the outcome?
5. Incomplete outcome data: were incomplete outcome data adequately addressed?
6. Selective outcome reporting: were reports of the study free of suggestion of selective outcome reporting?
7. Other sources of bias: was the study apparently free of other problems that could put it at a high risk of bias?

Each study received a judgment of low risk, high risk, or unclear risk of bias (indicating either lack of sufficient information to make a judgment or uncertainty over the risk of bias) for each of the seven domains. Studies were finally grouped into the following categories:

- *Low risk of bias* (plausible bias unlikely to seriously alter the results) if all key domains of the study were at low risk of bias.
- *Unclear risk of bias* (plausible bias that raises some doubt about the results) if one or more key domains of the study were unclear.
- *High risk of bias* (plausible bias that seriously weakens confidence in the results) if one or more key domains were at high risk of bias.

Prospective and retrospective studies were graded as low, moderate, or high risk of bias according to the following criteria, adapted from the Bondemark scoring system [17]:

- *Low risk of bias* (all criteria should be met):
  - Randomized clinical study or a prospective study with a well-defined control group.
  - Defined diagnosis and endpoints.
  - Diagnostic reliability tests and reproducibility tests described.
  - Blinded outcome assessment.
- *Moderate risk of bias* (all criteria should be met):
  - Cohort study or retrospective cases series with defined control or reference group.
  - Defined diagnosis and endpoints.
  - Diagnostic reliability tests and reproducibility tests described.
- *High risk of bias* (one or more of the following conditions):
  - Large attrition.
  - Unclear diagnosis and endpoints.
  - Poorly defined patient material.

The Grading of Recommendations Assessment, Development and Evaluation (GRADE) [16] was implemented to assess the overall quality of evidence for the studies included in this systematic review, according to which the overall evidence is rated as high, moderate, low, and very low. The outcomes included in GRADE were divided into categories regarding the different parameters that had been assessed in the primary studies.

- *High quality of evidence* implies that the true effect lies close to that of the estimate of the effect
- *Moderate quality of evidence* implies that the true effect is likely to be close to the estimate of the effect, but there is a possibility that it is substantially different
- *Low quality of evidence* implies that our confidence in the effect estimate is limited: the true effect may be substantially different from the estimate of the effect
- *Very low quality of evidence* implies that the true effect is likely to be substantially different from the estimate of effect.

## Results

### Study selection

**Fig. 1:**
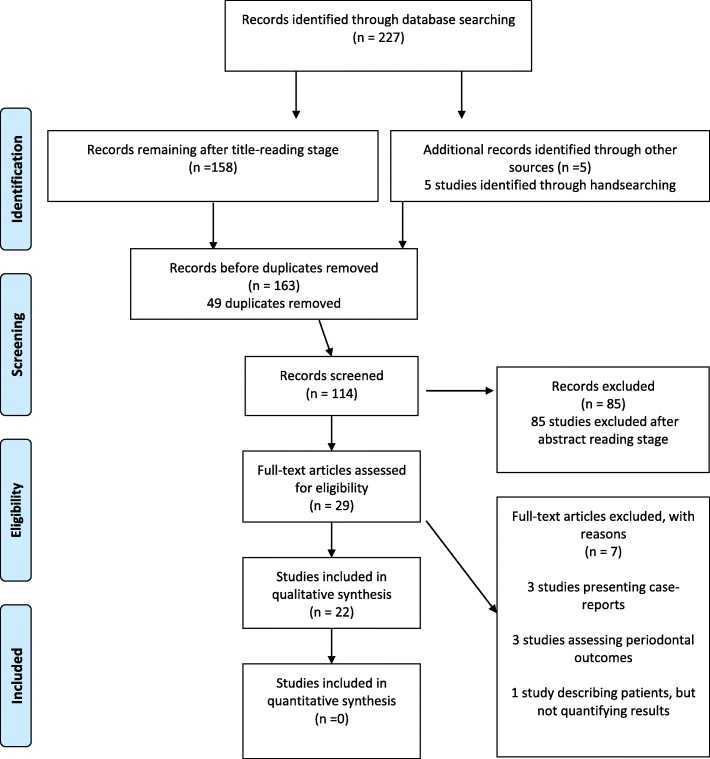
Studies’ flow diagram

The electronic search initially identified 227 relevant articles. One hundred fifty-eight papers remained after exclusion on the basis of title-reading. Five articles were added through hand-searching. After 49 duplicates’ removal, 114 papers were assessed for screening, and after abstract-reading, 85 studies were excluded leaving 29 articles to be read in full-text. After the application of specific inclusion and exclusion criteria, another seven articles were removed. In total, 22 studies were considered eligible for inclusion in the final analysis (Fig. 1).

### Study characteristics

The characteristics of each study are presented in detail in Table 1. Table 2 gives an overview of the results of the included studies regarding clinical parameters. Three studies [18–20] were RCTs, eight studies were of prospective [5, 21–27], and 11 of retrospective design [28–38].

**Table 1:** An overview of the included studies providing information on the experimental designs and settings

Author (year)	Title	Study design	Setting, treatment duration	Participants (number, sex)	Age of patients (mean age)	Inclusion criteria	Intervention group	Comparison group
Hennessy et al. [18] (2016)	A randomized clinical trial comparing mandibular incisor proclination produced by fixed labial appliances and clear aligners.	RCT	Setting: n/a Treatment duration: fixed appliance group, 11.3 months; clear aligner group, 10.2 months	44 patients (17M, 27F)	Invisalign group: 29.1 ± 7.5 years Fixed appliance group: 23.7 ± 7.0 years	- Age ≥ 18 years - No caries or periodontal disease - Mild Mn crowding (< 4 mm) - Non-extraction orthodontic treatment - Anteroposterior skeletal pattern within the average range (ANB 1–4^°^)	22 patients treated with Invisalign	22 patients treated with fixed appliances (self-ligating brackets)
Li et al. [19] (2015)	The effectiveness of the Invisalign appliance in extraction cases using the ABO model grading system: a multicenter randomized controlled trial.	RCT	2 orthodontic clinics at the Second Affiliated Hospital, Zhejiang University Invisalign treatment duration was 44% longer than fixed appliance treatment	152 patients (62M, 90F)	Invisalign group: 35.2 ± 7.3 years Fixed appliance group: 32.2 ± 8.3 years	- Patients aged ≥ 18 years - Extraction treatment - Patients consented to the research procedures and signed - Availability of pre- and post-treatment dental study models and panoramic films with good quality - Classified as being severe in complexity with a score of 25 using the discrepancy index (DI) of the ABO phase III clinical examination - Class I occlusion	76 patients treated with Invisalign	76 patients treated with fixed appliances
Bollen et al. [20] (2003)	Activation time and material stiffness of sequential removable orthodontic appliances. Part 1: Ability to complete treatment	RCT	University of Washington Regional Clinical Dental Research Center Primary endpoint: completion of initial aligners’ series	51 patients (15M, 36F)	34 years (range 19–55)	- Age ≥ 18 years - Ability to attend weekly appointments and to pay for services - Requirement for regular dental and periodontal maintenance program in case of caries or periodontal disease	51 patients randomly assigned to 4 intervention groups; either to hard/soft plastic appliance and 1 week/2 weeks activation time	The 4 groups were compared to each other
Solano-Mendoza et al. [21] (2016)	How effective is the Invisalign® system in expansion movement with Ex30′ aligners?	Prospective	Private clinic in Stuttgart, Germany Mean treatment duration: 657.4 ± 341.4 days	116 patients (46M, 70F)	36.57 ± 11.53 years	- Treatment with Ex30 aligner material - Expansion of the posterior upper teeth (from canine to 1st upper molar) - Presence of an initial and final digital model - Definition of the third palatal ruga - No presence of attachments on the initial or final model - No more than two models per patient	Expansion with Invisalign; 4 groups: (a) G1 (*n* = 40): expansion ≤ 1.99 mm in intermolar cuspid width (b) G2 (*n* = 45): expansion ≤ 3.99 mm (c) G3 (*n* = 14): patients subjected to expansion ≤ 5.99 mm (d) G4 (*n* = 10): expansion ≥ 6 mm. 7 patients unclassified due to current absence of one or both 1st molars	Initial and final virtual 3-D ClinCheck® models
Buschang et al. [26] (2015)	Predicted and actual end-of-treatment occlusion produced with aligner therapy	Prospective	1 private practice, Dallas, Texas, USA Treatment duration: n/a	27 patients (n/a)	n/a	Consecutive patients	27 consecutive patients treated with Invisalign	Final virtual 3-D ClinCheck® models
Castroflorio et al. [22] (2013)	Upper-incisor root control with Invisalign appliances	Prospective	2 private orthodontic clinics in a metropolitan area of northwest Italy Treatment duration: not reported	6 patients (2M, 4F)	26.3 ± 10.2 years	No patient had any record of anterior crossbite, anterior prosthodontic work, previous orthodontic treatment, craniofacial trauma, surgery, TMD, or orofacial pain	Invisalign patients (*n* = 6; 9 Mx incisors) needing palatal root torque as part of their treatment	Initial and final virtual 3-D ClinCheck® models for each upper incisor
Pavoni et al. [23] (2011)	Self-ligating versus Invisalign: analysis of dento-alveolar effects	Prospective	Department of Orthodontics “Tor Vergata,” Dental School, University of Rome Treatment duration: Invisalign group, 18 ± 2 months; self-ligating group, 18 ± 3 months	40 patients (19M, 21F) were equally divided into 2 groups: Invisalign® group (8M, 12F); self-ligating group (11M, 9F)	Invisalign group: 18.4 years Self-ligating group: 15.6 years	- Class I malocclusion - Mild crowding in Mn arch (mean: 4.4 ± 0.8 mm) - Permanent dentition - Vertebral maturation more advanced than CS4 (post-pubertal) - No previous orthodontic treatment	Invisalign + IPR (*n* = 20)	Fixed appliances (self-ligating; *n* = 20)
Kravitz et al. [5] (2009)	How well does Invisalign work? A prospective clinical study evaluating the efficacy of tooth movement with Invisalign	Prospective	Department of Orthodontics at the University of Illinois, Chicago Primary endpoint: completion of initial aligners’ series. The mean number of aligners per treatment was 10 Mx and 12 Mn with each aligner worn for 2–3 weeks	37 patients (14M, 23F)	31 years	- Age ≥ 18 years - Anterior crowding/spacing < 5 mm and adequate buccal interdigitation - Patients with posterior edentulous spaces were included if treatment did not entail space closure (1 participant had mandibular incisor extraction) - Clinicians were allowed to request/refuse IPR, proclination, attachments, and overcorrections on ClinCheck® - Only Invisalign attachments could be used and the tray could not be altered with scissors/thermopliers	37 patients/401 anterior teeth (198 Mx, 203 Mn) treated with Anterior Invisalign®	Final virtual 3-D ClinCheck® models
Kravitz et al. [24] (2008)	Influence of attachments and interproximal reduction on the accuracy of canine rotation with Invisalign	Prospective	Department of Orthodontics, University of Illinois, Chicago Mean duration: 7 months. Primary endpoint: completion of initial aligners’ series	31 patients (13M, 18F)	≥ 18 years	Same as Kravitz et al. [5] (2009)	31 patients/53 canines (33 Mx, 20 Mn) treated with anterior Invisalign® were divided in 3 groups: (a) attachments only (AO) (b) interproximal reduction only (IO) (c) neither attachments nor interproximal reduction (N)	Final virtual 3-D ClinCheck® models
Baldwin et al. [27] (2008)	Activation time and material stiffness of sequential removable orthodontic appliances. Part 3: Premolar extraction patients	Prospective	University of Washington Regional Clinical Dental Research Center Primary endpoint: completion of initial aligners’ series	24 patients (6M, 18F)	32.8 (range 18–54) years	Same as Bollen et al. [20] (2003) + at least 1 premolar extraction	24 patients treated with either hard/soft plastic appliance and 1 week/2 weeks activation time	No control group (pretreatment condition)
Vlaskalic and Boyd [25] (2002)	Clinical evolution of the Invisalign appliance	Prospective	University of the Pacific Mean treatment duration: group 1, 20 months; group 2, 27.2 months; group 3, 31.5 months	40 patients	14–52 years	- Fully erupted permanent dentition (except for 3rd molars) - Dental health with no immediate need for restorations - Availability for evening appointments - Desire to comply with orthodontic treatment	3 Invisalign groups based on severity of crowding: group 1 (*n* = 10 mild cases); group 2 (*n* = 15 moderate cases), and group 3 (*n* = 15 severe cases)	The 3 groups were compared to each other
Gu et al. [28] (2017)	Evaluation of Invisalign treatment effectiveness and efficiency compared with conventional fixed appliances using the Peer Assessment Rating index	Retrospective	Setting: Division of Orthodontics at Ohio State University College of Dentistry Treatment duration: Invisalign group, 13.35 months; fixed appliance group: 19.1 months	96 patients (34M; 62F)	Invisalign group: 26 ± 9.7 years Fixed appliances group: 22.1 ± 7.9 years	- Available pre- and posttreatment records—age ≥ 16 years - No auxiliary appliances other than elastics - Non-extraction patients - No orthognathic surgery or syndromic patients - Full permanent dentition except third molars	Invisalign (*n* = 48)	Fixed appliances (straight-wire edgewise appliances; *n* = 48)
Khosravi et al. [29] (2017)	Management of overbite with the Invisalign appliance	Retrospective	Setting: 3 private orthodontic offices; 2 located in the greater Seattle area, Wash and 1 in Vancouver, British Columbia Treatment duration: n/a	120 patients (36M; 84F)	33 years (interquartile range: 17)	- Age ≥ 18 years - 11 to 40 aligners used for each arch - A max use of 3 revision sets of aligners - Non-extraction treatment plan - No class II to class I occlusion change - Not significantly changed posterior-transverse relationships - No fixed appliances - Good-quality pre- and post-treatment cephalometric radiographs	Invisalign; stratified study sample as follows: 68 patients in the normal overbite group, 40 patients in the deep- bite group, and 12 patients in the open-bite group	The 3 groups were compared with each other
Houle et al. [30] (2016)	The predictability of transverse changes with Invisalign	Retrospective	Setting: Department of Preventive Dental Science, Division of Orthodontics, School of Dentistry, University of Manitoba - Orthodontic practice in Adelaide, Australia Treatment duration: 56 weeks	64 patients (23M, 41F)	31.2 years (range18–61 years)	- Age ≥ 18 years - Non-extraction treatment without any auxiliaries other than Invisalign attachments	Invisalign (*n* = 64)	Initial and final virtual 3-D ClinCheck® models
Ravera et al. [31] (2016)	Maxillary molar distalization with aligners in adult patients: a multicenter retrospective study.	Retrospective	Orthodontic private practices located in Torino (Italy) and Vancouver (Canada) Treatment duration: 24.3 ± 4.2 months	20 patients (9M, 11F)	29.73 ± 6.89 years	- Age ≥ 18 years old - Skeletal class I or II and a bilateral end-to-end molar relationship - Normodivergence on the vertical plane (SN-GoGn angle < 37°) - Mild crowding in the upper arch (≤ 4 mm) - Absence of mesial rotation of the upper 1st molar - Standardized treatment protocol, - Good compliance (wearing aligner time, ≥ 20 h per day) - Absence or previous extraction of the upper 3rd molars - Good quality radiographs	Invisalign (*n* = 20)	No control group
Duncan et al. [32] (2015)	Changes in mandibular incisor position and arch form resulting from Invisalign correction of the crowded dentition treated nonextraction	Retrospective	Single orthodontic practice Treatment duration: 1st group, 53.6 ± 21.1 weeks; 2nd group, 63.7 ± 20.7 weeks; 3rd group: 71.7 ± 16.3 weeks	61 patients (17M, 44F)	Adult patients (age n/a)	- Non extraction cases with or without IPR	3 intervention groups according to pre-treatment crowding of lower dentition (Carey’s analysis): (a) 20 mild (2.0–3.9 mm), (b) 22 moderate (4.0–5.9 mm), and (c) 19 severe (> 6.0 mm) cases	The 3 groups were compared to each other
Grünheid et al. [33] (2015)	Effect of clear aligner therapy on the buccolingual inclination of mandibular canines and the intercanine distance	Retrospective	University of Minnesota Mean treatment duration: Invisalign group, 13.4 ± 6.8 months; fixed appliance group: 20.2 ± 5.3 months	60 patients (30 in each group; 8M, 22F)	Invisalign group: 25 ± 11.8 years; fixed appliance group: 26.3 ± 13.5 years	- Fully erupted permanent dentition including incisors, canines, premolars, and 1st molars - Angle class I malocclusion with normal interarch molar relation - No periodontal attachment loss - Non-extraction orthodontic treatment - Pre- and posttreatment full-field of view CBCT scans - Both mandibular canines clearly visible in the CBCT scans	Invisalign (*n* = 30)	Fixed appliances (*n* = 30)
Simon et al. [34] (2014)	Treatment outcome and efficacy of an aligner technique – regarding incisor torque, premolar derotation and molar distalization	Retrospective	Private orthodontic practice in Cologne, Germany Treatment duration: n/a	30 patients (11M, 19F) initially, but 4 dropped out (*n* = 26)	32.9 ± 16.3 years Range 13–72 years	- Healthy patients - 1 of the 3 following tooth movements required: (1) Upper medial incisor torque > 10° (2) Premolar derotation > 10° (3) Molar distalization of an upper molar > 1.5 mm	3 Invisalign groups: (a) Incisor torque > 10° (b) Premolar derotation > 10° (c) Molar distalization > 1.5 mm. The groups were subdivided: in the 1st subgroup, movements were supported with an attachment, while in the 2nd subgroup no auxiliaries were used (except incisor torque, in which Power Ridges were used)	Initial and final virtual 3-D ClinCheck® models
Krieger et al. [35] (2012)	Invisalign® treatment in the anterior region. Were the predicted tooth movements achieved?	Retrospective	Setting and treatment duration: not reported	50 patients (16M, 34F)	33 ± 11.2 years	Frontal Mx and/or Mn crowding according to Little’s index of irregularity	Invisalign (*n* = 50)	Initial and final virtual 3-D ClinCheck® models
Krieger et al. [36] (2011)	Accuracy of Invisalign® treatments in the anterior tooth region. First results	Retrospective	Setting and treatment duration: n/a	35 patients (11M, 24F)	33 (range 15–59) years	- Orthodontic treatment exclusively with Invisalign - Consecutive post-treatment models and patient documentation - Presence of low-moderate Mx and/or Mn crowding	Orthodontic treatment exclusively with Invisalign (*n* = 35)	Initial and final virtual 3-D ClinCheck® models
Kuncio et al. [37] (2007)	Invisalign and traditional orthodontic treatment postretention outcomes compared using the American Board of Orthodontics Objective Grading System	Retrospective	Private practice in New York City Treatment duration: Invisalign group, 1.7 ± 0.8 years; fixed appliance group: 2.3 ± 0.8 years	22 patients (11 in each group; 1M, 10F)	34 years in the Invisalign group 26 years in the fixed applaince group	Non-extraction cases	Invisalign (*n* = 11)	Fixed appliances (*n* = 11)
Djeu et al. [38] (2005)	Outcome assessment of Invisalign and traditional orthodontic treatment compared with the American Board of Orthodontics objective grading system	Retrospective	Private practice in New York City Treatment duration: 1.4 years for the Invisalign® group; 1.7 years for the fixed appliance group	96 patients (gender n/a)	Invisalign®: 33.6 ± 11.8 years Fixed appliances: 23.7 ± 11.0 years	Non-extraction cases	Invisalign (*n* = 48)	Fixed appliances (*n* = 48)

*M* male, *F* female, *m.a* mean age, *Mx* maxillary, *Mn* mandibular, *IPR* interproximal reduction, *CBCT* cone-beam computed tomography, *n/a* not available

**Table 2:** Overview of the results, outcomes, and conclusions of the included studies

Author, year, design	Title	Subject group	Outcome assessed	Method of outcome assessment	Results	Conclusions
Hennessy et al. [18] (2016) RCT	A randomized clinical trial comparing mandibular incisor proclination produced by fixed labial appliances and clear aligners	Invisalign vs. fixed appliances	Mandibular incisor proclination produced by fixed appliances and Invisalign® aligners when treating patients with mild mandibular crowding	Comparison of pre-treatment and near-end treatment lateral cephalograms; the main outcome was the cephalometric change in mandibular incisor inclination to the mandibular plane at the end of treatment	- Mn incisor proclination: fixed appliances, 5.3 ± 4.3°; Invisalign®: 3.4 ± 3.2° (*P* > 0.05)	No difference in the amount of Mn incisor proclination produced by Invisalign® and fixed labial appliances in mild crowding cases
Li et al. [19] (2015) RCT	The effectiveness of the Invisalign appliance in extraction cases using the the ABO model grading system: a multicenter randomized controlled trial	Invisalign vs. fixed appliances	Treatment outcomes of the Invisalign® system by comparing the results of Invisalign® treatment with that of fixed appliances in class I adult extraction cases	The DI was used to analyze pretreatment records (study casts and lateral cephalograms) to control for initial severity of malocclusion. The ABO-OGS was used to systematically grade both pre- and post-treatment records	- Improved total mean scores of the OGS categories after treatment for both groups in terms of alignment, marginal ridges, occlusal relations, overjet, inter-proximal contacts, and root angulation - Invisalign® scores were significantly lower than fixed appliance scores for b-l inclination and occlusal contacts - Invisalign® had longer treatment duration (31.5 months) compared to fixed appliances (22 months)	Both Invisalign® and fixed appliances were successful in treating class I adult extraction cases, though Invisalign® required more time and showed worse performance in certain fields
Bollen et al. [20] (2003) RCT	Activation time and material stiffness of sequential removable orthodontic appliances. Part 1: Ability to complete treatment	Invisalign groups	Effects of activation time and material stiffness on the ability to complete the initial series of aligners, designed to fully correct each subject’s malocclusion	Initial PAR scores calculation, clinical evaluation and orthodontic records (progress study models and photographs) every 4 months	- 15/51 completed the initial regimen of aligners - 2 weeks activation interval more likely to lead to completion than 1 week (37% vs 21%) - No substantial differences between soft- and hard appliance in completion rate (27% vs 32%) - Highest completion rate (46%) for non-extraction and initial PAR score < 15 - Lowest completion rate (0%) in patients with ≥ 2 extractions	Greater likelihood for completion of the initial set of aligners for subjects with a non-extraction, 2 weeks activation regimen and low initial PAR scores
Solano-Mendoza et al. [21] (2016) Prospect.	How effective is the Invisalign® system in expansion movement with Ex30′ aligners?	Accuracy	A new method for measuring the predictability of expansion obtained by Invisalign® treatment and differences between the predicted (ClinCheck® models) and actual expansion at the end of treatment	Initial and final ClinCheck® virtual models measured with ToothMeasure® compared to initial and final actual 3D models measured with NemoCast® for evaluation of the following variables: canine gingival width, 1st premolar gingival width, 2nd premolar gingival width, 1st molar gingival width, canine cuspid width, 1st premolar cuspid width, 2nd premolar cuspid width, 1st molar cuspid width, canine depth, arch depth, 1st molar rotation, 1st right and left molar rotation, and 1st molar inclination	- Non-significant differences between the initial 3D models and ClinCheck® for all variables except for 1st molar cuspid width and arch depth - Statistically significant differences between the final 3D and ClinCheck® models for canine gingival width, 1st premolar gingival width, 2nd premolar gingival width, 1st molar gingival width, canine cuspid width, 1st premolar cuspid width, 2nd premolar cuspid width, 1st molar cuspid width	- Differences between the final 3D and ClinCheck® models showed that planned expansion at the end of treatment is not predictable
Buschang et al. [26] (2015) Prospect.	Predicted and actual end-of-treatment occlusion produced with aligner therapy	Accuracy	Differences between final actual models from the final virtual ClinCheck® models after treatment with Invisalign	Final ClinCheck® virtual models compared to final actual 3D models measured with MeshLab V1.30 software for evaluation of the American Board of Orthodontics (ABO) Objective Grading System (OGS)	Final virtual ClinCheck models showed significantly fewer overall OGS point deductions compared to final actual models (15 vs 24). Differences were mainly observed in alignment (1 vs 4 deductions), buccolingual inclinations (3 vs 4 deductions), occlusal contacts (2 vs 3 deductions), and occlusal relations (2 vs 4 deductions)	- The final virtual ClinCheck models do not accurately reflect the patients’ final occlusion, as measured by the OGS, at the end of active treatment
Castroflorio et al. [22] (2013) Prospect.	Upper-Incisor Root Control with Invisalign® Appliances	Accuracy	Efficiency of Align Technology’s Power Ridge in controlling the b-l inclination of upper incisors	ClinCheck® initial and final virtual setups for each upper incisor from the right and left default views compared to measurements on 3D-scans of actual dental models	- Mean torque values for the 9 upper incisors at T0: 20.9° on the virtual setups and 21.1° on the scanned casts - At T1, the torque values were 10.5° and 10.5°, respectively, and represented the torque prescription (10.4°)	- Invisalign® controls well the upper-incisor root torque, when a torque correction of about 10° is required
Pavoni et al. [23] (2011) Prospect.	Self-ligating versus Invisalign: analysis of dento-alveolar effects	Invisalign vs. fixed appliances	Dentoalveolar effects of the Invisalign® system and of self-ligating brackets treatment in relation to transverse dimension, arch perimeter and arch depth on Mx jaw	Measurements on pre- and post-treatment maxillary dental casts (intercanine-, interpremolar-, and intermolar width, arch depth, and arch perimeter)	-No significant differences in treatment duration. - Significant differences between the 2 groups with self-ligating causing further increases in the following variables as compared to Invisalign: intercanine width (cusp), 2.6 mm; first premolar width (fossa), 3.3 mm; first premolar width (gingiva), 2.3 mm; second premolar width (fossa), 2.0 mm; second premolar width (gingiva), 1.8 mm; arch perimeter, 1.3 mm	- Class I mild crowding can be treated by Invisalign® and self-ligating brackets at the same treatment duration - Invisalign® can easily tip crowns but not roots
Kravitz et al. [5] (2009) Prospect.	How well does Invisalign work? A prospective clinical study evaluating the efficacy of tooth movement with Invisalign	Accuracy	Differences between actual models and virtual ClinCheck® models in the anterior teeth, after treatment with Invisalign	DI scores (overjet, overbite, anterior open bite, and crowding) using a modified ABO-OGS on pretreatment digital models. Superimposition of virtual models of the predicted tooth position over the achieved tooth position (ToothMeasure®). Comparison between the predicted and achieved amount of tooth movement (i.e., expansion, constriction, intrusion, extrusion, mesiodistal tip, labiolingual tip, and rotation). Accuracy (%) = [(|predicted-achieved|/|predicted|) 100%]	- Invisalign® mean accuracy of tooth movement, 41% - Most accurate movement: lingual constriction (47.1%), least accurate movement: extrusion (29.6%; 18.3% for Mx and 24.5% for Mn central incisors), followed by mesio-distal tipping of the Mn canines (26.9%) - Canine rotation significantly less accurate than that of all other teeth, except for that of the Mx lateral incisors, especially at rotational movements > 15°. - Lingual crown tip significantly more accurate than labial crown tip - No statistical difference in accuracy between Mx and Mn for any movement on any specific tooth	-Further research is needed to understand the efficacy and biomechanics of the Invisalign® system -Prescription by clinicians should be made based on the patient’s treatment needs, while taking into account the limitations of the appliance
Kravitz et al. [24] (2008) Prospect.	Influence of attachments and interproximal reduction on the accuracy of canine rotation with Invisalign	Accuracy	Influence of attachments and IPR on canines undergoing rotational movement with Invisalign®	Tooth Measure® to compare the amount of canine rotations predicted with the ones achieved (in degrees). Accuracy (%) = [(|predicted-achieved|/|predicted|) 100%]	- Invisalign® mean accuracy of canine rotation was 35.8 ± 26.3% - No statistically significant difference in accuracy between the 3 groups - No statistically significant difference in rotational accuracy for Mx and Mn canines for any of the 3 groups - The vertical-ellipsoid was the most commonly prescribed attachment shape (70.5%)	The effectiveness of the Invisalign® system in canine derotation is limited and not significantly improved by vertical-ellipsoid attachments and IPR
Baldwin et al. [27] (2008) Prospect.	Activation time and material stiffness of sequential removable orthodontic appliances. Part 3: Premolar extraction patients	Invisalign only	Tipping of teeth adjacent to premolar extraction spaces during space closure with aligner appliances	Dental casts and panoramic radiographs pre-treatment and at the end of Invisalign treatment (potentially continued with fixed appliances)	- During treatment, the average radiographic changes in interdental angle were 21.5° (*P* < 0.0001; *n* = 10) in the mandible and 16.3° (*P* < 0.0001; *n* = 19) in the maxilla. On the models, the average changes were 20.8° (*P* < 0.0001; *n* = 12) in the mandible and 15.9° (*P* < 0.0001; *n* = 20) in the maxilla - No subject completed the initial series of aligners and only 1 ultimately completed treatment with aligners - The average time in the initial series of aligners before failure was 7 (range, 1–17) months and the average total time in aligners 16.6 (range, 6–28) months (treatment continued with fixed appliances)	- In premolar extraction patients treated with Invisalign, significant dental tipping occurs (it can be corrected with fixed appliances) - There is a trend for greater tipping of mandibular teeth into the extraction space and around second premolar extraction sites during treatment with aligners
Vlaskalic and Boyd [25] (2002) Prospect.	Clinical evolution of the Invisalign® appliance	Invisalign groups	Clinical evaluation of the Invisalign® system based on a feasibility study conducted in the University of the Pacific in 1997	Pre-, progress-, and post-treatment records including panoramic and lateral cephalometric radiographs, dental casts, intra-, and extraoral photographs.	Group 1: aligners need to be worn for at least 10 days each, patients tolerate aligners well, posterior open bite occurs in some patients, overcorrection of tooth position is necessary in initial 3-D setup Group 2: attachments are necessary for rotations of cylindrical shaped teeth, intrusion, extrusion, bodily tooth movement, extraction of teeth is possible Group 3: long vertical attachments are necessary from the start of treatment to maintain adequate root control in extraction cases, virtual tooth pontic system is esthetically and mechanically advantageous	-The Invisalign system is a viable alternative to conventional fixed and removable appliances - Patients in the permanent dentition with mild to moderate malocclusions may be greatly benefited when treatment is planned carefully - Further investigation is needed for the ultimate clinical potential of Invisalign®
Gu et al. [28] (2017) Retrosp.	Evaluation of Invisalign treatment effectiveness and efficiency compared with conventional fixed appliances using the Peer Assessment Rating index	Invisalign vs. fixed appliances	Effectiveness and efficiency of the Invisalign system compared with conventional fixed appliances in mild to moderate malocclusions	Comparison between patients treated with Invisalign® and fixed appliances assessing post-treatment PAR scores, post-treatment reduction in PAR scores, treatment duration, and malocclusion improvement	- Average pretreatment PAR scores: 20.81 for Invisalign and 22.79 for fixed appliances (NS) - Not statistically different posttreatment PAR scores and PAR score reduction between the 2 groups. - Invisalign® patients finished 5.7 months faster than those with fixed appliances (*P* = 0.0040). - All patients in both groups had > 30% reduction in PAR scores. - Odds of achieving “great improvement” in the Invisalign® group were 0.33 times greater than those in the fixed appliances group after controlling for age (*P* = 0.015)	- Both Invisalign® and fixed appliances are able to improve mild to moderate malocclusion - Fixed appliances were more effective than Invisalign in providing greater improvements - Treatment with Invisalign was finished on average 30% (5.7 months) faster than treatment with fixed appliances.
Khosravi et al. [29] (2017) Retrosp.	Management of overbite with the Invisalign appliance	Invisalign groups	Vertical dimension changes in patients with various pre-treatment overbite relationships treated only with Invisalign and other dental and skeletal changes	Pre- and post-treatment lateral cephalometric radiographs; cephalometric analyses by Dolphin Imaging, Chatsworth, Calif	- Deep bite patients had a median overbite opening of 1.5 mm, whereas the open bite patients had a median deepening of 1.5 mm. The median change for the normal overbite patients was 0.3 mm - Changes in incisor position were responsible for most of the improvements in the deep bite and open bite groups - Minimal changes in molar vertical position and mandibular plane angle	- Invisalign is relatively successful in managing overbite - Overbite is maintained in patients with normal overbite - Deep bite improvement primarily by proclination of Mn incisors - Invisalign corrects mild to moderate anterior open bites, primarily through incisor extrusion
Houle et al. [30] (2016) Retrosp.	The predictability of transverse changes with Invisalign	Accuracy	Differences between the initial and final actual models from the initial and final virtual ClinCheck® models after treatment with Invisalign, when planning transverse changes	- Comparison between pre- and posttreatment digital models, (created from an iTero scan) and digital models from Clincheck® (Align Technology) - Digital models were measured with Geomagic Qualify	- In the Mx, when dentoalveolar expansion was planned with Invisalign®, there was a mean accuracy of 72.8%: 82.9% at the cusp tips and 62.7% at the gingival margins, with prediction worsening toward the posterior region of the arch - For the Mn arch, there was an overall accuracy of 87.7%: 98.9% for the cusp tips and 76.4% for the gingival margins -Variance ratios for upper and lower arches were significantly different (*P* < 0.05)	- Clincheck® prediction of expansion involves more bodily movement of the teeth than that achieved clinically. More dental tipping was observed - Careful planning with overcorrection and other auxiliary methods of expansion may help reduce the rate of midcourse corrections and refinements, especially in the posterior region of the Mx
Ravera et al. [31] (2016) Retrosp.	Maxillary molar distalization with aligners in adult patients: a multicenter retrospective study	Invisalign group	Dentoalveolar and skeletal changes following maxillary molar distalization therapy with Invisalign in adult patients	Pre- and post-treatment lateral cephalometric radiographs	- Distal movement of the 1st molar: 2.25 mm without significant tipping and vertical movements - Distal movement of the 2nd molar: 2.52 mm without significant tipping (*P* = 0.056) and vertical movements - No significant movements on the lower arch. - SN-GoGn and SPP-GoGn angles showed no significant differences between pre- and post-treatment cephalograms	- Invisalign aligners are effective in distalizing Mx molars in selected end-to-end class II non-growing subjects without significant vertical and mesiodistal tipping movements - No changes to the facial height
Duncan et al. [32] (2015) Retrosp.	Changes in mandibular incisor position and arch form resulting from Invisalign correction of the crowded dentition treated nonextraction	Invisalign groups	Treatment outcomes in non-extraction cases with lower anterior crowding treated with Invisalign®	-Pre- and post-treatment records (digital study models and lateral cephalometric radiographs) -Cephalometric analysis to determine lower incisor changes - IPR and changes in arch width were also measured	- In the severe crowding group, there were statistically significant changes in lower incisor position and angulation - No significant differences in lower incisor position and angulation in the the mild and moderate crowding groups - Statistically significant increase in buccal expansion in all three groups.	- No change in the lower incisor position or angulation in mild to moderate lower anterior crowding cases -In non-extraction severe crowding cases (> 6 mm), the lower incisors tend to procline and protrude -Buccal arch expansion and IPR are important factors in crowding resolution -Intercanine, interpremolar, and intermolar widths do not differ among the three groups at post-treatment
Grünheid et al. [33] (2015) Retrosp.	Effect of clear aligner therapy on the buccolingual inclination of mandibular canines and the intercanine distance	Invisalign vs. fixed appliances	Treatment changes in b-l inclination of Mn canines and intercanine distance between patients treated with Invisalign® and conventional fixed appliances	Pre- and post-treatment CBCTs	- No significant pre-treatment difference between the groups regarding the b-l inclination of Mn canines and intercanine distance - Positive pre- and post-treatment b-l inclinations of Mn canines (i.e., their crowns were positioned buccal to their roots) for both groups - Significantly greater post-treatment b-l inclination in the Invisalign group - Significantly increased intercanine distance in the aligner group at the end of treatment	Invisalign seems to increase the Mn intercanine distance with little increase in b-l inclination compared to fixed appliances
Simon et al. [34] (2014) Retrosp.	Treatment outcome and efficacy of an aligner technique – regarding incisor torque, premolar derotation and molar distalization	Accuracy	Treatment efficacy of Invisalign® aligners for the following 3 predetermined tooth movements: incisor torque > 10°, premolar derotation > 10°, and molar distalization > 1.5 mm	- Comparison between the predicted amount of tooth movement by ClinCheck® and the amount achieved after treatment - Evaluation of the influence of auxiliaries (attachments/Power Ridge), the staging (movement/aligner), and the patient’s compliance with treatment	- Overall mean efficacy: 59 ± 0.2% - Mean accuracy for upper incisor torque: 42 ± 0.2% - Premolar derotation showed the lowest accuracy of approximately 40 ± 0.3% - Distalization of an upper molar was the most effective movement, with efficacy approximately 87 ± 0.2%	- Bodily tooth movement (molar distalization) can be effectively performed using Invisalign® aligners - Premolar derotation significantly depends on velocity and total amount of planed tooth movement - For upper incisor torque and premolar derotation, overcorrections/case refinements may be needed
Krieger et al. [35] (2012) Retrosp.	Invisalign® treatment in the anterior region. Were the predicted tooth movements achieved?	Accuracy	Differences in the anterior region between the initial and final actual models from the initial and final virtual ClinCheck® models after treatment with Invisalign	- Electronic digital caliper for measurements in casts - Evaluated parameters: upper/lower anterior arch length and intercanine distance, overjet, overbite, dental midline shift, and Little’s irregularity index - ClinCheck® was measured with ToothMeasure®	- Mx anterior crowding: initial, 5.4 (range 1.5–14.5) mm; final, 1.6 (range 0.0–4.5) mm - Mn anterior crowding: initial, 6.0 (range 2.0–11.5) mm; final, 0.8 (range 0.0–2.5) mm - Slight deviations between the initial actual and virtual ClinCheck® models in overjet (− 0.1 ± 0.3 mm), upper anterior arch length (− 0.3 ± 0.5 mm), lower anterior arch length (0.0 ± 0.5 mm), and in overbite (0.7 ± 0.9 mm)	- Moderate to severe anterior crowding can be successfully corrected with Invisalign® - Well predictable resolution of lower anterior crowding is achieved by protrusion of anterior teeth (i.e., enlargement of the anterior arch length) - In general, the achieved tooth movement was in accordance with the predicted movement for all parameters, except for overbite
Krieger et al. [36] (2011) Retrosp.	Accuracy of Invisalign® treatments in the anterior tooth region. First results	Accuracy	Differences between the initial and final actual models from the initial and final virtual ClinCheck® models after treatment with Invisalign	- Electronic dental caliper to measure pre- and post-treatment models - ToothMeasure® to measure the ClinCheck® - Examined parameters: overjet, overbite, and dental midline shift	- Slight deviations in overjet (0.1 ± 0.3 mm), overbite (0.3 ± 0.4 mm), and dental midline deviation (0.1 ± 0.4 mm) between initial actual and virtual models - Larger deviations in overjet (0.4 ± 0.7 mm), overbite (0.9 ± 0.9 mm), and dental midline shift (0.4 ± 0.5 mm) between final actual and virtual models	- Acceptable accuracy of Invisalign® technology during computerized transfer of malaligned teeth into the ClinCheck® presentation. - Tooth corrections in the vertical plane were more difficult to achieve. - Overcorrection in the final ClinCheck®, case refinement at treatment end or additional measures (e.g., horizontal beveled attachments or vertical elastics) are suggested to meet individualized therapeutic goals, especially in vertical corrections
Kuncio et al. [37] (2007) Retrosp.	Invisalign and Traditional Orthodontic Treatment Postretention Outcomes compared using the American Board of Orthodontics Objective Grading System	Invisalign vs. fixed appliances (retention)	Post-retention treatment outcomes in patients treated with Invisalign and those treated with traditional fixed appliances	- ABO-OGS analysis on panoramic radiographs and dental casts - Investigated parameters: total alignment, Mx anterior and posterior alignment, Mn anterior and posterior alignment, marginal ridges, b-l inclination, occlusal contacts, occlusal relations, overjet, interproximal contacts, root angulations - Evaluation after appliance removal (T1) and at a post-retention (T2) (3 years after appliance removal). - Efficacy in retention in comparison to Essix retainer after fixed appliances	- Post-retention worsening of total alignment and Mn anterior alignment for both groups - Higher post-retention changes in total alignment (ABO-OGS score) for Invisalign patients (− 2.9 ± 1.6) than patients treated with fixed appliances (− 1.4 ± 1.2) - Post-retention worsening of Mx anterior alignment in the Invisalign group only.	Greater relapse in the Invisalign® group for this observation period (approximately 3 years) for Invisalign than for fixed appliance group
Djeu et al. [38] (2005) Retrosp.	Outcome assessment of Invisalign and traditional orthodontic treatment compared with the American Board of Orthodontics objective grading system	Invisalign vs. fixed appliances	Treatment outcome of Invisalign compared to conventional fixed appliance treatment	- Pretreatment records (dental casts and lateral cephalograms) assessed with the DI (measurements: overjet, overbite, anterior open bite, lateral open bite, crowding, occlusion, lingual posterior crossbite, buccal posterior crossbite, cephalometrics, and other) - Posttreatment records (dental casts and panoramic radiographs) scored by ABO-OGS (measurements: alignment, marginal ridges, b-l inclination, occlusal contacts, occlusal relations, overjet, interproximal contacts, root angulation)	- Lower OGS passing rate for Invisalign® (27.1%) than that for fixed appliances - Invisalign® scores were significantly lower than fixed appliance scores for b-l inclination, occlusal contacts, occlusal relationships, and overjet (*P <* 0.05) - Invisalign® OGS scores negatively correlated to initial overjet, occlusion, and buccal posterior crossibite - Treatment duration on average 4 months shorter with Invisalign® than with fixed appliances (*P <* 0.05)	- Treatment results of fixed appliances are superior to those of Invisalign® (13 OGS points on average) - Reduced ability of Invisalign to correct large A-P discrepancies and occlusal contacts

*Prospect.*, prospective, *Retrosp.*, retrospective, *DI* discrepancy index, *ABO* American Board of Orthodontics, *OGS* Objective Grading System, *Mx* maxilla (or maxillary), *Mn* mandible (or mandibular), *NS* not statistically significant, *b-l* buccolingual

### Quality analysis

The quality assessment of the 22 studies is shown in Tables 3 and 4.

**Table 3:** Quality assessment of the included RCT studies

Author-year of publication	Study design	Sequence generation (selection bias)	Allocation concealment (selection bias)	Blinding of participants and personnel (performance bias)	Blinding of outcome assessors (detection bias)	Incomplete outcome data (attrition bias)	Selective reporting (reporting bias)	Other sources of bias	Overall risk
Hennessy et al. [18] (2016)	RCT	Aj: Low risk Sfj: Although not explicitly stated, sequence generation is very likely due to reference of random picking up of sealed opaque envelopes	Aj: Low risk Sfj: Sealed opaque, envelopes	Aj: Low risk Sfj: Incomplete blinding, but the review authors judge that the outcome is not likely to be influenced by lack of blinding	Aj: Low risk Sfj: No blinding of outcome assessment, but the review authors judge that the outcome measurement is not likely to be influenced by lack of blinding	Aj: Low risk Sfj: Missing outcome data balanced in numbers across intervention groups, with similar reasons for missing data across groups	Aj: Low risk Sfj: The study protocol is available and all of the study’s pre-specified outcomes that are of interest in the review have been reported in the pre-specified way	Aj: Low risk Sfj: The study appears to be free of other sources of bias	Low
Li et al. [19] (2015)	RCT	Aj: Low risk Sfj: Use of a computer random number generator	Aj: Low risk Sfj: Sequentially numbered, opaque, sealed envelopes	Aj: Low risk Sfj: Blinding ensured and unlikely that the blinding could have been broken	Aj: Low risk Sfj: Blinding of outcome assessment ensured and unlikely that the blinding could have been broken	Aj: Low risk Sfj: No missing outcome data	Aj: Low risk Sfj: The study protocol is available and all of the study’s pre-specified outcomes that are of interest in the review have been reported in the pre-specified way	Aj: Low risk Sfj: The study appears to be free of other sources of bias	Low
Bollen et al. [20] (2003)	RCT	Aj: Low risk Sfj: Reference to a random number list	Aj: Low risk Sfj: Randomization schedule based on a list of random numbers performed by a calibrated investigator, unaware of the treatment plan	Aj: Low risk Sfj: Incomplete blinding, but the review authors judge that the outcome is not likely to be influenced by lack of blinding	Aj: Low risk Sfj: No blinding of outcome assessment, but the review authors judge that the outcome measurement is not likely to be influenced by lack of blinding	Aj: Low risk Sfj: Missing outcome data balanced in numbers across intervention groups, with similar reasons for missing data across groups	Aj: Low risk Sfj: The study protocol is available and all of the study’s pre-specified outcomes that are of interest in the review have been reported in the pre-specified way	Aj: Low risk Sfj: The study appears to be free of other sources of bias	Low

*Aj:* authors’ judgment, *Sfj* support for judgment

**Table 4:** Quality assessment of the included prospective and retrospective studies

Author-year of publication	Study design and defined control group	Adequately defined patient material	Defined diagnosis and end points	Diagnostic reliability and reproducibility tests	Blinded outcome assessment	Overall risk
Solano-Mendoza et al. [21] (2016)	+ (prospective)	+	+	+	−	Moderate
Buschang et al. [26] (2015)	+ (prospective)	+	+	+	−	Moderate
Castroflorio et al. [22] (2013)	+ (prospective)	−	−	−	−	High
Pavoni et al. [23] (2011)	+ (prospective)	+	+	+	−	Moderate
Kravitz et al. [5] (2009)	+ (prospective)	+	+	−	−	High
Kravitz et al. [24] (2008)	+ (prospective)	+	+	−	−	High
Baldwin et al. [27] (2008)	- (prospective, uncontrolled)	+	−	+	+	High
Vlaskalic and Boyd [25] (2002)	+ (prospective)	+	−	−	−	High
Gu et al. [28] (2017)	+ (retrospective)	+	+	+	+	Moderate
Khosravi et al. [29] (2017)	+ (retrospective)	+	+	+	−	Moderate
Houle et al. [30] (2016)	+ (retrospective)	+	+	+	−	Moderate
Ravera et al. [31] (2016)	+ (retrospective)	+	+	+	+	Moderate
Duncan et al. [32] (2015)	+ (retrospective)	+	+	+	−	Moderate
Grünheid et al. [33] (2015)	+ (retrospective)	+	+	+	+	Moderate
Simon et al. [34] (2014)	+ (retrospective)	+	+	−	−	High
Krieger et al. [35] (2012)	+ (retrospective)	+	+	+	−	Moderate
Krieger et al. [36] (2011)	+ (retrospective)	+	+	+	−	Moderate
Kuncio et al. [37] (2007)	+ (retrospective)	+	+	+	+	Moderate
Djeu et al. [38] (2005)	+ (retrospective)	+	+	+	−	Moderate

#### RCTs

The three RCTs [18–20] were judged to be at an overall low risk of bias, due to the low risk of bias that applied to each domain based on the Cochrane risk of bias tool [16] (Table 3).

#### Prospective studies

Three prospective studies [21, 26, 35] were graded as moderate and five [5, 22, 24, 25, 27] as high risk of bias. Although they were all studies of prospective design, no blinding in relation to outcome assessment was reported in all except one [27] study, which also lacked control, among other limitations (Table 4).

#### Retrospective studies

Ten out of the 11 identified retrospective studies [28–38] were graded as moderate risk of bias, since all the pre-determined criteria were met. Only one retrospective study [34] was of high risk of bias, because it did not include any diagnostic reliability and reproducibility tests (Table 4).

### Qualitative synthesis of the included studies

#### Study settings

An overview of the experimental design of the included studies is presented in Table 1. Eight studies [5, 21, 22, 24, 30, 34–36] used patients’ virtual ClinCheck® models of the predicted tooth movement as control group, aided by ToothMeasure® [5, 21, 22, 24, 34–36] or Geomagic Qualify [30], in order to investigate the treatment’s efficacy. More specifically, the extent that the initial and final actual models were different from the initial and final virtual models after treatment was evaluated. However, two of them had similar samples and outcomes with two other studies, namely [5] with [24, 35] with [36]. We decided not to exclude any of these studies, since additional information was provided. Seven studies [18, 19, 23, 28, 33, 37, 38] compared treatment outcome of Invisalign® orthodontic treatment with that of conventional fixed appliances. At last, four studies [20, 25, 29, 32] compared Invisalign® groups to each other, while one study [31] did not have any control or comparison group.

All studies tested mainly non-growing patients, and most of them included patients of an average age of 30 years [5, 19–21, 29–31, 34–38]. Non-extraction cases were used as study samples in nine studies [18, 28–33, 37, 38]. Treatment duration differed among and within studies, as expected according to malocclusion severity and the implemented intervention. Six studies [18, 22, 29, 34–36] did not report on treatment duration. Finally, only one study [37] reported post-retention treatment outcomes by comparing the induced changes in patients treated with Invisalign® with those treated with traditional fixed appliances. The evaluation was conducted at a maximum post-retention time of 3 years after appliance removal, with all the patients undergoing at least 1 year of retention.

#### Clinical findings

Table 2 gives an overview of the results of the included studies regarding clinical parameters, grouped in the following three subject categories.

##### A. Accuracy

The accuracy of Invisalign® was reported in nine studies [5, 21, 22, 24, 26, 30, 34–36], where it was evaluated as the deviation between the achieved and the planned tooth movements. The findings among studies were varying ranging from sufficient accuracy in resolving anterior crowding [35, 36] and distalizing maxillary molars [34] to contradictory findings in upper incisor root control [22, 34] and to inadequacies in bodily expansion of the maxillary posterior teeth [21, 26, 30], canine [5, 24] and premolar [34] rotational movements, extrusion of maxillary incisors5, and in overbite control [35, 36].

##### B. Invisalign® vs traditional fixed appliances

Seven studies [18, 19, 23, 28, 33, 37, 38] compared Invisalign® orthodontic treatment outcomes to that of conventional fixed appliances. A recent RCT study [18] found no significant difference in the amount of mandibular incisor proclination produced by Invisalign® and fixed labial appliances in mild crowding cases, supported by a retrospective study [23], which also concluded that treatment duration in these cases was similar for the two methods, though Invisalign was not so successful in root alignment. Gu et al. [28] reported similar outcomes, but shorter duration with Invisalign, for mild to moderate malocclusions. However, worse performance of Invisalign was noted in more severe cases, a finding also supported by Djeu et al. [38]. In the same line, in a RCT study, Li et al. [19] concluded that both therapeutic approaches can succeed in class I adult extraction cases, though Invisalign required more time and was less able to correct bucco-lingual inclination and occlusal contacts. The latter findings are also in agreement with those of two retrospective studies [33, 38].

Differences between the two methods in post-retention alterations were investigated in one retrospective moderate risk of bias study [37]. Greater relapse was found 1–3 years posttreatment after Invisalign® treatment compared to conventional orthodontic therapy with fixed appliances.

##### C. Invisalign groups only

In an early exploratory study, Vlaskalic and Boyd [25] concluded that Invisalign® may be more beneficial for patients in the permanent dentition with mild to moderate malocclusions after careful treatment planning. Another early exploratory RCT study [20] also concluded that non-extraction treatment of milder malocclusions has greater chances to be successfully treated by Invisalign.

Three recent retrospective studies also tested various Invisalign groups. One showed the moderate ability of Invisalign to manage overbite [29]. More specifically, normal overbite was well maintained, but deep bite was partially corrected, through mandibular incisor proclination. Open bite was also partially corrected, but mainly through incisor extrusion. On the other hand, a second study [31] reported the ability of Invisalign to bodily distalize maxillary molars in adult nonextraction mild class II cases (≤ ½ cusp), with no changes in facial height. Finally, a third study [32] showed the ability of Invisalign to correct mild to moderate crowding nonextraction cases without causing significant changes in the mandibular incisor position and inclination. On the contrary, such changes (protrusion and proclination) were induced in cases with severe crowding (≥ 6 mm).

The Grading of Recommendations Assessment, Development and Evaluation (GRADE) [16] was implemented to assess the overall quality of evidence for the studies included in this review and for outcomes that were assessed by two or more studies. GRADE tables illustrate the outcomes that were assessed by two or more studies (Additional file 1, 2, and 3). 

### Quantitative synthesis of the included studies

The lack of standardized protocols impeded a valid interpretation of the actual results through pooled estimates. Substantial differences in the implemented interventions, participants’ characteristics (age and gender distribution), treatment duration, and investigated outcomes indicated significant methodological heterogeneity. Therefore, a meta-analysis was not feasible.

## Discussion

In order to successfully deliver orthodontic treatment, clinicians need to carefully plan an appropriate therapeutic approach based on the current scientific evidence. Although this is not the only determining factor for the final decision, as clinical experience and patient’s opinion also play an important role, this information needs to be taken into consideration to assess the possibilities and limitations of each treatment modality.

With regard to Invisalign®, to date, there are four systematic reviews available, pertaining to clinical effects of the system [12–15], with one of them [14] evaluating periodontal health issues. Given the limited available evidence in certain earlier attempts [12, 15] and the evaluation of the effectiveness of Invisalign® under the wider spectrum of clear aligners [13, 15], strong conclusions regarding the investigated clinical efficiency of the Invisalign® system were not feasible. This ambient obscurity on a highly increasing treatment approach was the reason to perform a systematic search of the literature and assess the available scientific evidence with respect to the clinical outcomes of the Invisalign® orthodontic treatment. Due to the relatively unexplored topic, an attempt was made to conduct the present systematic review to a high standard, in order to minimize any chance of bias, but also include all the available information.

Indeed, a considerable number of studies were included in this review, though only three of them were RCTs [18–20], with low risk of bias. From the remaining 19 studies, 8 were of prospective [5, 21–27] and 11 of retrospective design [28–38] with moderate [21, 23, 26, 28–38] or high [5, 22, 24, 25, 27, 34] risk of bias. Thus, since it was difficult to assess the outcomes and reach safe results and conclusions, a strict methodology in both the data extraction and quality analysis was attempted. The methodological quality of the retrieved studies was thoroughly evaluated and a qualitative synthesis of the results was performed.

Considerable differences in participants’ characteristics, types of interventions, reporting of clinical outcomes, and treatment’s duration was evident, thus, preventing the implementation of a meta-analysis. More specifically, the number of patients recruited ranged from 6 [22] to 152 [19], which indicates a strong methodological difference among the study protocols and in strength of the stated results. Concerning the age of the patients that underwent treatment with Invisalign®, it varied between 13 [34] and 61 [30] years, with all studies primarily including non-growing patients, most of them having an average age of 30 years [5, 19–21, 29–31, 34–38], and most of them with moderate [21, 29–31, 35–38] and high [5, 34] risk of bias. This reveals a strong lack of information for growing individuals and indicates that Invisalign® is at present a preferred treatment option for late adolescent and adult patients, who usually have higher esthetic demands.

With regard to the outcome measures, measurements in pre- and post-treatment records were made. The records included the following: actual or/and digital dental casts [5, 19–23, 25, 28, 30, 32, 35–38], panoramic radiographs [25, 37, 38], lateral cephalograms [18, 19, 25, 29, 31, 32, 38], CBCTs [33], and photographs [19, 20, 25, 38]. The discrepancy index (DI) and the peer assessment rating index (PAR) were used in the pre-treatment records to assess the initial severity of malocclusion [5, 19, 28, 38]. The American Board of Orthodontics – Objective-grading system (ABO-OGS) was used in three studies [5, 19, 38] to systematically grade both pre- and post-treatment records evaluating various clinical parameters. ToothMeasure®, which is the Invisalign®’s proprietary superimposition software, was also used to make measurements on 3D dental models, including the initial and final ClinCheck® virtual models [5, 24, 35, 36].

As for the overall treatment duration, there were different completion criteria and varying outcomes among and within studies. When compared to conventional appliances, the Invisalign® system showed significantly shorter treatment duration in three studies [28, 33, 38], while no difference was reported in another study [23]. All these studies evaluated nonextraction treatment of mild to moderate malocclusions and scored as moderate risk of bias. On the contrary, one study on extraction treatment reported longer duration for Invisalign treatment [19], with low risk of bias. Thus, it seems that Invisalign might treat faster mild nonextraction cases, but it requires more time than fixed appliance treatment for more complex cases.

Substantial variation in the investigated clinical outcomes was noted among studies. The majority of them focused on the accuracy of Invisalign® or its comparison to conventional fixed appliances. The first was found sufficient when certain malocclusion features, such as overjet or anterior arch length discrepancy, were tested [35, 36] or for maxillary molar distalization [34]. The efficacy on maxillary molar distalization (≤ ½ cusp) was also supported by another clinical study [31]. However, important limitations were reported for bodily expansion of the maxillary posterior teeth [21, 30], canine [5, 24] and premolar [34] rotational movements, extrusion of maxillary incisors 5, and in overbite control [35, 36]. All of these referred studies scored as moderate according to Bondemark scoring system [17]. Based on these findings, the use of additional attachments or overcorrections was commonly suggested in the literature for these types of movement. As for the comparison to fixed appliances, from studies with moderate [23, 28] to low [18] risk of bias, it seems that Invisalign performs well in mild to moderate non-extraction cases [18, 23, 28], but it cannot equally succeed in more difficult cases, including extraction cases [19, 27, 28, 33, 38]. Teeth inclinations and occlusal contacts seem to be among the major limitations of Invisalign [19, 33, 38], most of them judged as moderate [23, 33, 38] risk of bias and only two with low [18, 19]. The results from studies that included only different Invisalign groups are in agreement with the abovementioned findings [20, 25, 29, 32].

In addition, only one study [37], graded as moderate, included a post-treatment observational period investigating the stability of treatment outcomes with Invisalign®, indicating a general lack of information with regard to retention. Although the amount of evidence is limited, this study showed more relapse in the Invisalign cases, as compared to fixed appliance treatment, that might be attributed to the inadequacies in obtaining certain bodily movements and solid occlusal contacts.

Overall, evidence was of moderate quality. Apart from the three RCTs [18–20], where a low risk of bias was considered, the remaining prospective and retrospective studies were graded as moderate [21, 23, 26, 28–38] or high [5, 22, 24, 25, 27, 34] risk of bias. The studies’ review showed high amount of heterogeneity in terms of methodology and outcome reporting that impeded a valid interpretation of the actual results through pooled estimates. However, there was substantial consistency among researchers that the Invisalign® system is a viable alternative to conventional orthodontic therapy in correcting mild to moderate malocclusions, without extractions. Moreover, when the treatment is carefully planned, Invisalign® aligners can safely straighten dental arches in terms of leveling and derotating the teeth, except for canines and premolars. Finally, crown tipping can be easily performed. On the other hand, important limitations include arch expansion through bodily tooth movements, extraction space closure, corrections of occlusal contacts, and larger antero-posterior and vertical discrepancies.

All things considered, it is evident that more high-quality research of prospective design with respect to the clinical outcomes of Invisalign® needs to be carried out in the future. A standardized methodology including control samples would be valuable in obtaining comparative results with conventional approaches. Furthermore, though more than half of the studies included in the present review have been published in the last 5 years (range 2012–2017), the findings of the review should be interpreted with some caution; the continuous improvement of the Invisalign system (especially in 2013 with SmartTrack® material) [39] may not allow for direct synthesis and valid comparisons between older studies with the most recent ones, as the inclusion of data from different iterations of Invisalign material may become a factor of bias. This is, of course, a major consideration when synthesis of studies’ results for clinical evidence is concerned, in an era that software, scanners, and 3D printer costs are more affordable and potential in-house printing of aligners is rapidly growing. Last but not least, the long-term effectiveness pertaining to retention outcomes also needs further investigation, whereas complete lack of evidence is evident for growing patients.

## Conclusions

Despite the fact that orthodontic treatment with Invisalign® is a widely used treatment option, apart from non-extraction treatment of mild to moderate malocclusions of non-growing patients, no clear recommendations about other indications of the system can be made, based on solid scientific evidence.

Although this review included a considerable number of studies, treatment outcomes need to be interpreted with caution due to the high heterogeneity. Further research with parallel arm RCTs or well-designed prospective trials are needed to form robust clinical recommendations for a wide spectrum of malocclusions and for growing patients.

Albeit the existing limitations, the following conclusions were made, based on the available evidence:

- Invisalign might treat faster mild non-extraction cases, but it requires more time than fixed appliance treatment for more complex cases.
- Invisalign® aligners can safely straighten dental arches in terms of leveling and derotating the teeth (except for canines and premolars, where a small inadequacy was reported). Crown tipping can be easily performed.
- Teeth inclinations and occlusal contacts seem to be among the limitations of Invisalign®, when accuracy of planned movements achieved with aligners is concerned.
- Use of additional-novel attachments might be more effective for various types of movement, such as bodily expansion of the maxillary posterior teeth, canine and premolar rotational movements, extrusion of maxillary incisors, and in overbite control.

### Additional files

Additional file 1: GRADE Working Group grades of evidence. Summary of findings: Invisalign compared in groups of different treatment modalities or divergent severity of crowding. (DOCX 16 kb)

Additional file 2: GRADE Working Group grades of evidence. Summary of findings: Accuracy of treatment result. (DOCX 16 kb)

Additional file 3: GRADE Working Group grades of evidence. Summary of findings: Invisalign compared to fixed appliances in adults. (DOCX 16 kb)

## Appendix

**Table 5:** Search strategy, Medline via PubMed, 28 August 2017

1. invisalign	158
2. invisalign [tiab]	158
3. clear aligner	48
4. aligner* AND orthodont*	181
5. ortho caps	5
6. orthocaps	1
7. invisible AND orthodont*	69
8. removable AND aligner	33
9. esthetic AND splint AND orthodont*	75
10. transparent* AND orthodont*	63

## Abbreviations

ABO-OGS: American Board of Orthodontics – Objective-grading system
GRADE: Grading of Recommendations Assessment, Development and Evaluation
RCT: Randomized clinical trial
