# Supplementary material for: Clinical effectiveness of Invisalign® orthodontic treatment: a systematic review
Source: Prog Orthod. 2018 Sep 28;19:37. doi: 10.1186/s40510-018-0235-z (PMC6160377; doi:10.1186/s40510-018-0235-z)
Supplement: Supplementary file 1 — GRADE Working Group grades of evidence. Summary of findings: Invisalign compared in groups of different treatment modalities or divergent severity of crowding. (DOCX 16 kb) [file 40510_2018_235_MOESM1_ESM.docx]

**Summary of findings:**

# Invisalign compared in groups of different treatment modalities or divergent severity of crowding

**Patient or population**: adults **Setting**:

**Intervention**: Invisalign **Comparison**:

| Outcomes | Impact | | № of participants  (studies) | | Certainty of the evidence (GRADE) | |
| --- | --- | --- | --- | --- | --- | --- |
| Treatment in mild to moderate malocclusions assessed with: pre and post treatment records | | One study reported limited compliance in extraction cases. Two studies reported faster completion of treatment with aligners when the malocclusion was mild to moderate. | | 91  (3 observational studies) 1,2,3 | | ⨁◯◯◯  VERY LOW  a,b,c,d,e |
| Treatment in mild to moderate malocclusions  assessed with: par score calculation, photographs and study models | | This study showed that there was a greater likeligood for completion of therapy for non-extraction cases with 2-week activation protocol. | | 51 (1 RCT) ^4^ | | ⨁⨁⨁⨁  HIGH |

***The risk in the intervention group** (and its 95% confidence interval) is based on the assumed risk in the comparison group and the **relative effect** of the intervention (and its 95% CI).

**CI:** Confidence interval

**GRADE Working Group grades of evidence**

**High certainty:** We are very confident that the true effect lies close to that of the estimate of the effect

**Moderate certainty:** We are moderately confident in the effect estimate: The true effect is likely to be close to the estimate of the effect, but there is a possibility that it is substantially different

**Low certainty:** Our confidence in the effect estimate is limited: The true effect may be substantially different from the estimate of the effect

**Very low certainty:** We have very little confidence in the effect estimate: The true effect is likely to be substantially different from the estimate of effect

**Explanations**

1. no control group to one stuudy
2. absence of reliability tests, and undefined diagnosis in one study, whereas all studies didn't have blinded outcome assessment
3. different samples and subgroups between studies, leading to heterogenity in results
4. indirectness due to substantial differences between the population, the intervention, or the outcomes measured in relevant research studies e. variability in sample age and gender was not equally distributed among subgroups

**References**

1. 30. Duncan LO, Piedade L,Lekic M,Cunha RS,Wiltshire WA.. Changes in mandibular incisor position and arch form resulting from Invisalign correction of the crowded dentition treated nonextraction. . Angle Orthod.; 2016.
2. Vlaskalic V, Boyd RL. Clinical evolution of the Invisalign® ap-pliance.. J Calif Dent Assoc; 2002.
3. Baldwin DK, King G,Ramsay DS,Huang G,Bollen AM.. Activation time and material stiffness of sequential removable orthodontic appli-ances. Part 3: premolar extraction patients.. Am J Orthod Dentofacial Orthop; 2008 .
4. Bollen AM, Huang G,King G et a.. Activation time and material stiffness of sequential removable orthodontic appliances. Part 1: Abil-ity to complete treatment.. Am J Orthod Dentofacial Orthop.; 2003.
