# Supplementary material for: Clinical effectiveness of Invisalign® orthodontic treatment: a systematic review
Source: Prog Orthod. 2018 Sep 28;19:37. doi: 10.1186/s40510-018-0235-z (PMC6160377; doi:10.1186/s40510-018-0235-z)
Supplement: Supplementary file 2 — GRADE Working Group grades of evidence. Summary of findings: Accuracy of treatment result. (DOCX 16 kb) [file 40510_2018_235_MOESM2_ESM.docx]

**Summary of findings:**

# Accuracy of treatment result

**Patient or population**: accuracy **Setting**:

**Intervention**: Invisalign

**Comparison**: software prognosed occlusion

| Outcomes | Impact | | № of participants  (studies) | | Certainty of the evidence (GRADE) | |
| --- | --- | --- | --- | --- | --- | --- |
| Accuracy in rotations  assessed with: Invisalign software | | One study reported that premolar derotation was the least accurate with 40 % (+-0,3%) accuracy. The other two studies showed mean accuracy of canine rotation of 35,8% (+-26,3%), which was the least accurate in comparison to rotations of other teeth. | | 94  (3 observational studies) 1,2,3 | | ⨁◯◯◯  VERY LOW  1,2,3,a,b |
| Expansion of posterior maxillary teeth assessed with: Invisalign software | | One study showed that the accuracy overall was 72,8% and 82,9% at the casp tips. The second study reported that the expansion planned by the final virtual models was not predictable. | | 143  (2 observational studies) ^4,5^ | | ⨁◯◯◯  VERY LOW ^b,c^ |
| Accuracy in incisor torque control assessed with: Invisalign software | | The first study showed 51,5% accuracy in incisor torque. The second study showed insignificant differences between virtual and actual measurements, confirming the accuracy of Invisalign. | | 32  (2 observational studies) ^1,6^ | | ⨁◯◯◯  VERY LOW  b,c,d,e |

***The risk in the intervention group** (and its 95% confidence interval) is based on the assumed risk in the comparison group and the **relative effect** of the intervention (and its 95% CI).

**CI:** Confidence interval

**GRADE Working Group grades of evidence**

**High certainty:** We are very confident that the true effect lies close to that of the estimate of the effect

**Moderate certainty:** We are moderately confident in the effect estimate: The true effect is likely to be close to the estimate of the effect, but there is a possibility that it is substantially different

**Low certainty:** Our confidence in the effect estimate is limited: The true effect may be substantially different from the estimate of the effect

**Very low certainty:** We have very little confidence in the effect estimate: The true effect is likely to be substantially different from the estimate of effect

**Explanations**

1. high risk of bias to three of studies , due to lack of blinded outcome assessment and diagnostic tests
2. indirectness due to substantial differences between the population, the intervention, or the outcomes measured in relevant research studies c. heterogenity between the samples of the studies
3. high risk of bias in both studies, due to lack of blinded outcome assessment and diagnostic tests
4. due to limited sample size, wide Confidence Intervals

**References**

1. Simon M, Keilig L,Schwarze J,Jung BA,Bourauel C.. Treatment out-come and efficacy of an aligner technique—regarding incisor torque, premolar derotation and molar distalization. BMC Oral Health; 2014.
2. al, Kravitz,et. Influence of attachments and inteproximal reduction on the accuracy of canine rotation with Invisalign. Angle Orthodontist; 2008.
3. al., Kravitz,et. How well does Invisalign work? A prospective clinical study evaluating the efficacy of tooth movement with Invisalign. American Journal of Orthodontics and Dentofacial Orthopedics; 2009.
4. Houle JP, Piedade L,Todescan R Jr,Pinheiro FH.. The predictability of transverse changes with Invisalign. . Angle Orthod.; 2017.
5. Solano-Mendoza B, Sonnemberg B,Solano-Reina E,Iglesias-Linares A.. How effective is the Invisalign® system in expansion movement with Ex30' aligners? . Clin Oral Investig.; 2017.
6. al, Castroflorio,et. Upper-Incisor Root Control with Invisalign Appliances. J Clin Orthod; 2003.
