# Supplementary material for: Clinical effectiveness of Invisalign® orthodontic treatment: a systematic review
Source: Prog Orthod. 2018 Sep 28;19:37. doi: 10.1186/s40510-018-0235-z (PMC6160377; doi:10.1186/s40510-018-0235-z)
Supplement: Supplementary file 3 — GRADE Working Group grades of evidence. Summary of findings: Invisalign compared to fixed appliances in adults. (DOCX 16 kb) [file 40510_2018_235_MOESM3_ESM.docx]

**Summary of findings:**

# Invisalign compared to fixed appliances in adults

**Patient or population**: adults **Setting**:

**Intervention**: Invisalign

**Comparison**: fixed appliances

| Outcomes | Impact | | № of participants  (studies) | | Certainty of the evidence (GRADE) | |
| --- | --- | --- | --- | --- | --- | --- |
| Buccolingual inclination  assessed with: pre- and near-end treament cephalograms | | One study showed that there was greater post-treatment buccolingual inclination in the Invisalign group, whereas the other two studies reported that fixed appliances were more effective in improving buccolingual inclination. | | 196  (3 observational studies) 1,2,3 | | ⨁◯◯◯  VERY LOW ^a^ |
| Treatment duration assessed with: study casts | | Two studies reported faster treatment completion with Invisalign (4 to 5,7 months) when compared to conventional appliances. The third study reported not significant difference between Invisalign and self-ligating brackets. | | 232  (3 observational studies) 1,3,4 | | ⨁◯◯◯  VERY LOW ^b^ |
| Buccolingual inclination  assessed with: pretreatment and near-end treatment cephalograms | | One study showed that change in incisor proclination was 3,4 +-3,2 degrees for tretament with Invisalign, while for fixed appliances the change was 5,3+- 4,3 degrees. The second study , accoridng to ABO-OGS, demonstrated that Invisalign scores were significantly lower(-3,55points) than fixed appliances scores(-5,85). | | 196  (2 RCTs) ^5,6^ | | ⨁⨁⨁⨁  HIGH |
| Treatment duration assessed with: study casts | | In this study, Invisalign had longer treatment duration (9,5 months). | | 44 (1 RCT) ^5^ | | ⨁⨁⨁◯  MODERATE ^c^ |

***The risk in the intervention group** (and its 95% confidence interval) is based on the assumed risk in the comparison group and the **relative effect** of the intervention (and its 95% CI).

**CI:** Confidence interval

**GRADE Working Group grades of evidence**

**High certainty:** We are very confident that the true effect lies close to that of the estimate of the effect

**Moderate certainty:** We are moderately confident in the effect estimate: The true effect is likely to be close to the estimate of the effect, but there is a possibility that it is substantially different

**Low certainty:** Our confidence in the effect estimate is limited: The true effect may be substantially different from the estimate of the effect

**Very low certainty:** We have very little confidence in the effect estimate: The true effect is likely to be substantially different from the estimate of effect

**Explanations**

1. heterogenity between samples of studies-number
2. indirectness due to substantial differences between the intervention samples, because in one study fewer than 50% of patients complete their treatment c. small sample of patients

**References**

1. Pavoni, C.,Lione,R. Laganà,P. Cozza.. Self-ligating versus Invisalign: analysis of dento-alveolar effects. . Ann Stomatol (Roma); 2011.
2. Grünheid T, Gaalaas S,Hamdan H,Larson BE.. Effect of clear aligner therapy on the buccolingual inclination of mandibular canines and the intercanine distance.. Angle Orthod.; 2016.
3. Djeu G, Shelton C,Maganzini A.. Outcome assessment of Invisalign and traditional orthodontic treatment compared with the American Board of Orthodontics objective grading system.. Am J Orthod Dentofacial Orthop. ; 2005.
4. Gu J, Tang JS,Skulski B,Fields HW Jr,Beck FM,Firestone AR et al.. Evaluation of Invisalign treatment effectiveness and efficiency com-pared with conventional fixed appliances using the Peer Assessment Rating index. . Am J Orthod Dentofacial Orthop.; 2017.
5. Hennessy J, Garvey T,Al-Awadhi EA.. A randomized clinical trial com-paring mandibular incisor proclination produced by fixed labial appli-ances and clear aligners.. Angle Orthod. ; 2016.
6. Li W, Wang S,Zhang Y.. The effectiveness of the Invisalign appliance in extraction cases using the the ABO model grading system: a multi-center randomized controlled trial.. Int J Clin Exp Med. ; 2015.
